# Clinical Scenarios in Hospitalized Patients With Hemophilia

**DOI:** 10.7759/cureus.106284

**Published:** 2026-04-01

**Authors:** Jose C Alvarez-Payares, Edwin J Ariza Parra, Adriana Margarita María Trejos Tenorio, Ioka De La Peña Lozano, Daniel Ribero-Vargas

**Affiliations:** 1 Hematology, Clínica Astorga, Medellín, COL; 2 Hematology, Instituto de Cancerología, Clínica Las Américas, Medellín, COL; 3 Internal Medicine, Hospital Quirón Salud, Torrevieja, ESP; 4 General Medicine, Hospital Pablo Tobón Uribe, Medellín, COL; 5 Internal Medicine, Clínica CES, Medellín, COL

**Keywords:** antithrombotic therapy, bleeding, bypass therapy, cardiovascular disease, factor replacement, hemophilia, inhibitors, perioperative management

## Abstract

Hemophilia is an X-linked recessive bleeding disorder caused by a deficiency of coagulation factor VIII (hemophilia A) or factor IX (hemophilia B). Despite therapeutic advances that have extended life expectancy, hospitalized patients with hemophilia present complex clinical challenges. This narrative review, illustrated with four structured clinical cases, addresses the most common scenarios encountered in the management of hospitalized patients with hemophilia: acute bleeding, perioperative management without inhibitors, perioperative management with inhibitors, and antithrombotic therapy in patients with comorbid cardiovascular disease.

Factor replacement remains the cornerstone of therapy, with target levels and dosing duration determined by bleeding site and procedure type. Inhibitor development occurs in up to 30% of hemophilia A patients and necessitates the use of bypassing agents (recombinant activated factor VII or activated prothrombin complex concentrate). Perioperative management requires a multidisciplinary approach with 24-hour laboratory and blood bank availability. Cardiovascular comorbidities, including ischemic heart disease and atrial fibrillation, occur at rates comparable to the general population in aging patients with hemophilia; antithrombotic therapy must be individualized according to baseline factor levels, bleeding phenotype, and inhibitor status. Left atrial appendage closure represents a viable alternative to long-term anticoagulation in selected high-risk patients.

Optimal management of hospitalized patients with hemophilia demands individualized, multidisciplinary care. Clinicians should be familiar with factor replacement protocols, inhibitor detection and management strategies, and the growing challenge of balancing bleeding risk with antithrombotic therapy in an aging hemophilia population. These practical considerations are essential for hospital-based clinicians involved in the care of this complex patient group.

## Introduction and background

Hemophilia is a congenital X-linked recessive bleeding disorder affecting almost exclusively males, with women generally presenting as asymptomatic carriers [1]. It is a disorder of secondary hemostasis caused by partial or total deficiency of coagulation factor VIII (F8 gene; hemophilia A) or factor IX (F9 gene; hemophilia B) [1]. Disease severity is classified according to residual factor activity as mild (6-40 IU/dL), moderate (1-5 IU/dL), or severe (<1 IU/dL) (Table 1) [2]. The hallmark clinical feature is spontaneous or trauma-induced bleeding - including mucocutaneous, musculoskeletal, intracavitary, and intracranial hemorrhage - which may be life-threatening (Table 1) [2].

**Table 1:** Classification of hemophilia severity by factor activity This table is original and was created by the authors based on the information presented in this reference [2]; no previously published content was reused.

Severity	Factor Activity (UI/dL)	Bleeding Risk
Mild	>5–40	Severe bleeding with major trauma or surgery
Moderate	1–5	Occasional spontaneous bleeding; severe bleeding with trauma or surgery
Severe	<1	Frequent spontaneous bleeding (joints and muscles)

Globally, an estimated 1,125,000 individuals are affected by hemophilia, with a prevalence of approximately 17.1 and 3.8 per 100,000 males for hemophilia A and B, respectively [3]. Females account for fewer than 10% of cases (approximately 3% in hemophilia A and 6% in hemophilia B), and severe disease occurs in approximately 6.0 and 1.1 per 100,000 persons, respectively [3]. Regarding causes of hospitalization, up to 74% of admissions are related to minor bleeding events, 13% involve major bleeding, and the remainder are attributable to elective orthopedic procedures [4].

Therapeutic advances have substantially improved outcomes in hemophilia; however, life expectancy remains below that of the general population. A population-based study conducted in the Netherlands between 2001 and 2018 demonstrated that mortality rates in persons with hemophilia exceeded those of the general population across all age groups, with a standardized mortality ratio greater than 1 for both hemophilia A and B, and that intracranial hemorrhage and HIV-related complications remained leading causes of death [4]. Nevertheless, survival has improved considerably over recent decades, largely driven by the widespread adoption of prophylactic factor replacement and advances in inhibitor management [4]. As a result, the clinical profile of hospitalized patients with hemophilia has evolved, now encompassing the acute management of age-related comorbidities in addition to bleeding events [4]. Admissions now frequently involve the acute management of age-related comorbidities - including hypertension, dyslipidemia, and type 2 diabetes - rather than bleeding episodes alone [5]. This growing complexity has considerable implications for clinical decision-making and healthcare resource utilization. The economic burden is substantial, encompassing direct treatment costs, losses in labor productivity, and intangible costs related to pain, suffering, and psychosocial impact on patients and their families [6]. Quality of life and life expectancy remain directly linked to access to treatment, a challenge that disproportionately affects patients in low- and middle-income countries [6].

Hemophilia is universally recognized as a high-cost condition, given that coagulation factor concentrates account for more than 70% of hospitalization costs compared with laboratory and imaging studies [7]. This burden escalates considerably in patients who develop inhibitory antibodies, in whom treatment costs may be up to threefold higher [7,8]. In many health systems - particularly in low- and middle-income countries - hemophilia is classified as a rare or orphan disease, with corresponding legal frameworks mandating special access to necessary health technologies; however, implementation remains inconsistent and access inequitable across regions [9].

This article provides a practical, evidence-based narrative review of the four principal clinical scenarios encountered in the management of hospitalized patients with hemophilia, with updated guidance for each. The clinical cases presented are fictitious and constructed on the basis of the authors' clinical experience. The literature discussed in each clinical scenario was identified through a non-systematic search of PubMed/MEDLINE, using the following MeSH terms and keywords: "hemophilia," "factor VIII," "factor IX," "acute bleeding," "perioperative management," "inhibitors," "bypassing agents," "emicizumab," "cardiovascular disease," "antithrombotic therapy," and "atrial fibrillation." Priority was given to clinical practice guidelines and prospective studies published within the last 10 years.

## Review

Clinical scenarios

In hospitalized patients with hemophilia, clinicians must determine whether bleeding is life-threatening; whether a surgical procedure is required; and whether comorbid conditions necessitate pharmacological therapy that increases bleeding risk [2]. Classification of both bleeding severity and the presence or absence of inhibitors is essential, as these factors dictate the degree of factor replacement required [2]. Four clinical scenarios and their principal management guidelines are presented below.

Scenario 1: Hemophilia Patient With Acute Bleeding

Case scenario:A 29-year-old male with severe hemophilia A (weight 70 kg), with mild hemorrhagic phenotype on factor prophylaxis, presents with two weeks of sudden-onset left iliac fossa pain, atraumatic, without macroscopic bleeding or peritoneal irritation. Ultrasound reveals a possible hematoma in the left pelvic cavity. What is the appropriate management approach?

Severe or life-threatening bleeding events should be suspected clinically and confirmed with laboratory testing and imaging [10]. These events include central nervous system (CNS) bleeding, ocular hemorrhage, muscle bleeding with neurovascular compromise (including iliopsoas hematoma), gastrointestinal or intra-abdominal hemorrhage, potential airway compromise, prolonged or anemia-producing bleeding, and bleeding secondary to high-energy trauma [10]. Severe life-threatening bleeding is most common in severe hemophilia, but is not exclusive to it [10].

The most common bleeding manifestation is hemarthrosis of the knee, elbow, ankle, hip, or shoulder [2], characterized by acute pain, inflammatory changes, and restricted range of motion. Management requires factor replacement, analgesics (non-steroidal anti-inflammatory drugs (NSAIDs) may be used cautiously in selected patients), rest, ice, and avoidance of weight-bearing on the affected limb [11]. Arthrocentesis should be performed only when factor levels exceed 100 IU/dL in order to prevent septic arthritis, neurovascular compromise, or compartment syndrome [11].

The primary treatment goal is preventing and controlling severe bleeding by raising factor levels to safe hemostatic targets, as established by the World Federation of Hemophilia (WFH) guidelines [12]. Dosing and duration depend on the bleeding site and hemophilia type (Table 2) [12]. When a severe or potentially life-threatening bleed is suspected, factor replacement must be initiated as promptly as possible, without delay for diagnostic confirmation. Medium-term goals include preventing recurrence and minimizing end-organ complications [12].

**Table 2:** Recommended plasma factor levels by bleeding site HA: Hemophilia A; HB: Hemophilia B. This table is original and was created by the authors based on the information presented in this reference [12]; no previously published content was reused.

Site of Bleeding	HA: Target Factor Levels (UI/dL)	HA: Duration (days)	HB: Target Factor Levels (UI/dL)	HB: Duration (days)
Hemarthrosis	40–60	1–2	40–60	1–2
Superficial muscle	40–60	2–3	40–60	2–3
Iliopsoas/Deep muscle	Initial: 80–100, Maintenance: 30–60	1–2, 3–5	Initial: 60–80, Maintenance: 30–60	1–2, 3–5
Intracranial	Initial: 80–100, Maintenance: 50	1–7, 8–21	Initial: 50–80, Maintenance: 20–40	1–3, 8–14
Throat/Neck	Initial: 80–100, Maintenance: 50	1–7, 8–14	Initial: 60–80, Maintenance: 30	1–7, 8–14
Gastrointestinal/Intra-abdominal	Initial: 80–100, Maintenance: 50	7–14, 7–14	Initial: 60–80, Maintenance: 30	7–14, 7–14
Renal	50	3–5	40	3–5
Deep laceration	50	5–7	40	5–7

Treatment planning must account for each factor's half-life and incremental recovery. The half-life of factor VIII is approximately 12 hours (requiring administration every 12 hours), and that of factor IX is 18-24 hours (allowing once-daily dosing) [2]. Dose calculation is based on body weight and expected recovery: for FVIII, each 1 IU/kg raises plasma activity by approximately 2 IU/dL; for FIX, each raises by approximately 1 IU/dL [2]. Accordingly:

FVIII dose (IU) = 0.5 × body weight (kg) × desired level (IU/dL)

FIX dose (IU) = 1.0 × body weight (kg) × desired level (IU/dL)

Case resolution: This young man with hemophilia A presents with an acute pelvic hematoma - a potentially life-threatening bleed requiring immediate factor VIII replacement [12]. Hemodynamic stability must be monitored; the degree of anemia must be assessed to guide transfusion support; and factor activity must be measured [12]. Per WFH guidelines, the initial factor VIII target is 80-100 IU/dL for 7-14 days, followed by a maintenance target of 50 IU/dL [12]. The initial dose is calculated as 0.5 × 70 kg × 90 IU/dL = 3,150 IU, adjusted to the nearest available vial size (typically 500-1,000 IU per vial) [12].

Scenario 2: Perioperative Management Without Inhibitors

Case scenario: A 57-year-old male (weight 80 kg) with severe hemophilia A, on prophylaxis with recombinant FVIII (rFVIII) for six years, presents with hemophilic arthropathy of elbows, knees, and ankles. He is admitted for pulmonary nodule resection via thoracoscopy. Physical examination reveals hypertrophied, non-tender knees. Recent laboratory results show FVIII activity of 2%, with no inhibitors. What is the most appropriate management approach?

In the hemophilia patient undergoing surgery, the following must be considered: urgency (elective versus emergency), procedure type (Table 3), disease severity based on factor activity (Table 1), and presence or absence of inhibitors, as these factors collectively determine intraoperative and postoperative bleeding risk [13,14]. It is important to note that patients currently receiving extended half-life (EHL) factor concentrates or non-factor therapies such as emicizumab may require specific perioperative adaptations, as these agents typically necessitate supplementation with standard factor replacement when surgery is performed [15].

**Table 3:** Surgical procedure classification HA: Hemophilia A; HB: Hemophilia B. This table is original and was created by the authors based on the information presented in this reference [14]; no previously published content was reused.

Criterion	Major Procedure	Minor Procedure
Definition	Access to the body cavity, removal of an internal organ, opening of a fascial plane, crossing a mesenchymal barrier (pleura, peritoneum, dura), and anatomical distortion from prior surgery	Invasive procedure manipulating connective tissue, skin, or mucous membranes
Preoperative factor target (UI/dL)	HA: 80–100; HB: 60–80	HA and HB: 50–80
Duration of factor therapy	>7 days	<7 days

Before any procedure, factor levels must be optimized - targeting 100 IU/dL for major surgery and ≥60 IU/dL for minor procedures. If levels at the time of induction are below target, additional replacement must be administered preoperatively [13]. Intraoperative hemostatic monitoring with thromboelastography (TEG) or rotational thromboelastometry (ROTEM) is recommended to guide real-time management [16].

Elective procedures in patients with hemophilia require a multidisciplinary approach encompassing hematology, surgery, anesthesiology, continuous laboratory monitoring, and pharmacy coordination for factor availability. The following evidence-based recommendations apply [13,14]. The hemophilia diagnosis and treatment history must be documented at the time of surgical scheduling, and procedures should be scheduled during morning hours whenever possible to ensure full laboratory and pharmacy support throughout the day [13]. Factor concentrate availability must be confirmed in the hospital pharmacy for perioperative and postoperative use, and blood products must be reserved as needed [13]. Inhibitor status must be verified before any procedure; if inhibitors are present, management should follow the principles outlined in Scenario 3 [14]. The patient's hemorrhagic phenotype and prior hemostatic response to factor replacement should be reviewed [14]. For postoperative analgesia, acetaminophen and opioids are preferred; intramuscular injections must be avoided [13]. All antiplatelet agents should be discontinued at least one week before surgery, and herbal supplements at least three days before [13]. Factor concentrates should be initiated one hour before surgical incision, with subsequent dosing guided by intraoperative factor activity measurements [14]. Tranexamic acid is both safe and effective as an adjuvant hemostatic agent for major and minor procedures, without increasing thrombotic risk [13]. If neuraxial anesthesia is planned, factor levels must reach ≥50 IU/dL before needle placement [14]. For patients receiving EHL concentrates, both one-stage clot-based and chromogenic FVIII assays should be obtained preoperatively, as discordance between the two has been documented; the lower result should guide perioperative management [15].

In the postoperative period, target factor levels should exceed 60 IU/dL for hemophilia A and 40 IU/dL for hemophilia B during the first three postoperative days [14]. Factor activity should be measured every 12 hours and adjusted according to clinical status [13]. For thromboprophylaxis, intermittent pneumatic compression devices are preferred; early ambulation and physical therapy should be initiated as soon as clinically feasible [17].

Case resolution: This patient with hemophilia A without inhibitors is scheduled for major elective thoracic surgery, with low residual factor activity and high intraoperative bleeding risk. Preoperative workup should include a complete blood count, coagulation studies, and viral serologies (hepatitis B virus (HBV), hepatitis C virus (HCV), and human immunodeficiency virus (HIV)) [13]. Factor concentrate availability must be confirmed with the hospital pharmacy, and adjuvant hemostatic agents (tranexamic acid) should be prepared [13]. Surgery should be scheduled as early in the morning as possible [13].

Initial preoperative FVIII dose: 0.5 × 80 kg × 100 IU/dL = 4,000 IU.

Scenario 3: Perioperative Management With Inhibitors

Case scenario: A 30-year-old woman with severe hemophilia A (on prophylaxis with rFVIII for 12 years) is admitted for thyroid mass resection. Recent laboratory results show FVIII activity of 1% and inhibitor titer of 25 Bethesda units (BU). What is the appropriate management approach?

Patients with hemophilia may develop neutralizing alloantibodies (IgG) directed against exogenous factor VIII or IX [2]. Inhibitors are more prevalent in patients of Hispanic or African ancestry with severe disease, occurring in up to 30% of hemophilia A patients and approximately 10% of hemophilia B patients; approximately 80% of cases emerge within the first 20 exposure days to factor concentrate [18,19]. Inhibitors should be clinically suspected in previously treated patients who experience worsening bleeding despite adequate factor replacement [18]. This complication carries significant morbidity and mortality, and annual or semiannual inhibitor screening is recommended for all patients receiving factor concentrates (Table 4) [20]. The 2024 International Society on Thrombosis and Haemostasis (ISTH) guideline recommends that emicizumab prophylaxis be considered as the preferred strategy over bypassing agents in high-responding inhibitor patients, and that all surgical procedures in inhibitor-positive patients be performed exclusively at expert hemophilia treatment centers [21].

**Table 4:** Characteristics of hemophilia patients with inhibitors aPCC: Activated prothrombin concentrate complex, BU: Bethesda units; FIX: Factor IX; GI: Gastrointestinal; rFVIIa: Recombinant VII activated factor; rFVIIIa: Recombinant VIII activated factor. This table is original and was created by the authors based on the information presented in this reference [19]; no previously published content was reused.

Feature	Hemophilia A	Hemophilia B
Incidence	~30% severe; 5%–10% mild/moderate	<5%; exclusively severe disease
Risk factors	Hispanic/African ancestry; high-intensity factor exposure	Family history; null mutations
Complications	Increased hospitalization and mortality; mucocutaneous, urogenital, and GI bleeding	Anaphylaxis (50%); nephrotic syndrome with FIX exposure
Prophylaxis	rFVIIIa; emicizumab	rFVIIa or aPCC (if no allergy/anaphylaxis)
Bleeding management	Low responder (<5 BU): higher factor doses; high responder (>5 BU): bypassing agents (rFVIIa or aPCC)	
Inhibitor monitoring	Every 6–12 months after factor initiation, then annually	

Patients are classified as low responders (<5 BU) or high responders (>5 BU) based on inhibitor titer [18]. In low responders, inhibitory antibodies are often transient and typically disappear within six months without specific intervention [18]. In high responders, titers may decline over time but rise rapidly upon re-exposure to factor concentrate due to an anamnestic immune response [18]. Active inhibitor surveillance is therefore recommended every 6-12 months, with particular attention prior to surgical procedures and following intensive factor exposure [22].

In patients with inhibitors, in addition to the general measures outlined in Scenario 2, perioperative management requires higher factor doses in low responders and the use of bypassing agents in high responders, such as recombinant activated factor VII (rFVIIa) or activated prothrombin complex concentrate (aPCC/FEIBA) [20,23]. These recommendations are guideline-based (WFH 3rd edition) and supported by expert consensus, given the limited availability of prospective controlled trial data in this population [20]. Patients receiving emicizumab who require aPCC for breakthrough bleeding or perioperative hemostasis are at increased risk of thrombotic microangiopathy (TMA), pulmonary embolism, and ischemic events, particularly at high aPCC doses (>50 IU/kg) or with cumulative daily doses exceeding 100 IU/kg [24]. For this reason, rFVIIa is the preferred bypassing agent in patients on emicizumab prophylaxis [25]. If prolonged perioperative aPCC use is unavoidable for major elective surgery, discontinuation of emicizumab several months in advance, accounting for its elimination half-life of approximately 30 days, should be considered in consultation with the treating hematologist [24].

Dosing guidance for rFVIIa is 90 µg/kg preoperatively, then every two hours for two days (minor surgery) or five days (major surgery), followed by every six hours until wound healing is confirmed [22]. aPCC is administered at 50-100 IU/kg before surgery, continued every 6-12 hours with a maximum daily dose of 200 IU/kg [22].

Case resolution: This patient with severe hemophilia A and a high inhibitor titer (25 BU; high responder) is scheduled for major elective surgery. Bypassing agent therapy is indicated per ISTH guidelines [21]. As she is not currently receiving emicizumab, the choice between rFVIIa and aPCC depends on institutional availability and clinician experience. Hemostatic monitoring will be performed using TEG or a thrombin generation assay [15].

Scenario 4: Hemophilia Patient Requiring Antiplatelet or Anticoagulant Therapy

Case scenario: A 55-year-old male with severe hemophilia A (on prophylaxis with rFVIII for 10 years and hemophilic arthropathy of elbows, knees, and ankles) is hospitalized for non-ST elevation acute coronary syndrome (NSTEMI) and new-onset non-valvular atrial fibrillation (AF). He has recently been diagnosed with hypertension and type 2 diabetes. Recent laboratory results show a FVIII baseline activity of 2%, with no inhibitors. What is the appropriate management approach?

Although hemophilia predisposes to bleeding, affected patients may develop acute conditions such as acute coronary syndrome (ACS), AF, or venous thromboembolism (VTE) requiring antiplatelet agents, anticoagulants, or both [26]. The incidence of cardiovascular disease among aging persons with hemophilia is rising and may affect up to 15% of patients, representing a growing clinical challenge that demands individualized, multidisciplinary management [26]. Therapeutic decisions must be made collaboratively by a team including hematology, cardiology, and - when applicable - vascular surgery. Table 5 summarizes evidence-based and guideline-informed considerations for antithrombotic therapy according to cardiovascular condition [27,28].

**Table 5:** Antithrombotic therapy according to cardiovascular condition in patients with hemophilia ASA: Acetylsalicylic acid; BU: Bethesda units; DAPT: Dual antiplatelet therapy (ASA + P2Y12 inhibitor); DOAC: Direct oral anticoagulant; LAA: Left atrial appendage; SAPT: Single antiplatelet therapy. This table is original and was created by the authors based on the information presented in this reference [24,25]; no previously published content was reused.

Condition	Drug/Intervention	Factor Target	Duration
Ischemic heart disease	Percutaneous coronary intervention (PCI)	80–100 UI/dL	Minimum 48 hours
Ischemic heart disease	Cardiac surgery	80–100 UI/dL	Until the wound healed
Ischemic heart disease	DAPT (ASA + P2Y12 inhibitor)	≥30 UI/dL	4 weeks
Ischemic heart disease	SAPT (ASA)	≥5 UI/dL	Indefinite
Atrial fibrillation	DOAC (if CHA₂DS₂-VASc ≥ 2 and basal factor ≥ 30 UI/dL)	≥30 UI/dL	Indefinite
Atrial fibrillation	LAA closure + DAPT	≥80 UI/dL (procedure); ≥30 UI/dL (DAPT)	Procedure: 48 hours; DAPT: 4 weeks
Atrial fibrillation	LAA closure + SAPT (ASA)	≥80 UI/dL (procedure); ≥5 UI/dL (SAPT)	Procedure: 48 hours; SAPT: 8 weeks

The lifetime prevalence of ischemic heart disease in patients with hemophilia may reach 19.5%, with an estimated prevalence of 15% in those older than 60 years [26]. PCI is preferred over thrombolysis due to the high bleeding risk associated with systemic fibrinolysis in this population [27]. Factor levels > 80 IU/dL are required before the procedure [17]. Loading doses of aspirin and clopidogrel are administered, along with a single weight-based dose of unfractionated heparin (70-100 U/kg) immediately before PCI [28]. DAPT is recommended for four weeks, with factor levels maintained at ≥20-30 IU/dL, followed by indefinite single antiplatelet therapy (aspirin) with factor levels ≥ 5 IU/dL [28]. The 2023 EHA-ISTH-EAHAD-ESO Clinical Practice Guidance further specifies that a minimum trough factor level of 1-5 IU/dL is acceptable during single antiplatelet therapy and at least 20 IU/dL during DAPT, with individualization based on bleeding phenotype as the guiding principle [29].

AF prevalence in patients with hemophilia parallels that of the general population, increasing with age and estimated at 3.4% in those older than 60 years [30]. Hemophilia does not appear to confer protection against thromboembolic complications in patients with AF [26]. Anticoagulation decisions are guided by stroke risk (CHA₂DS₂-VASc score) and bleeding risk based on residual factor activity; the HAS-BLED score is insufficient in this context as it does not incorporate hemophilia severity or inhibitor status [31]. When anticoagulation is indicated, direct oral anticoagulants (DOACs) at low doses are preferred over vitamin K antagonists (VKAs) due to a superior safety profile and lower risk of intracranial hemorrhage [31]. In patients with both high cardioembolic and high bleeding risk in whom long-term anticoagulation is contraindicated, LAA closure is a safe and effective alternative, with outcomes comparable to oral anticoagulation [31]. The 2023 EHA-ISTH-EAHAD-ESO guidance explicitly states that providing long-term prophylactic factor replacement solely to enable anticoagulation for AF is not considered feasible or appropriate; LAA closure, therefore, represents the preferred strategy in patients with severe hemophilia and high stroke risk who cannot sustain safe factor levels for anticoagulation [29].

Although less common, venous thrombotic complications may occur in patients with hemophilia, particularly in the context of surgery, prolonged immobilization, or supratherapeutic factor levels [32]. Factor levels > 150 IU/dL have been associated with a 4.8-fold increased risk of thrombosis compared with levels < 100 IU/dL [33]. Provoked VTE in patients with hemophilia is generally treated for six to eight weeks rather than the 12-week standard for the general population, reflecting the competing hemorrhagic risk [32].

Key clinical considerations when prescribing antithrombotic therapy in patients with hemophilia include the following. Clinicians should assess the frequency and severity of prior bleeding episodes (spontaneous versus trauma-provoked), inhibitor status, and the patient's documented response to factor replacement [27]. Anticoagulation is contraindicated when the risk of uncontrolled bleeding outweighs the expected benefit [27]. When anticoagulation is indicated, agents with short half-lives and readily available reversal strategies are preferred, such as unfractionated heparin or low molecular weight heparin, rather than fondaparinux [28]. Anticoagulation and DAPT may be used with relative safety in patients on factor prophylaxis achieving trough levels > 30 IU/dL, though all decisions must be individualized [28]. Antiplatelet agents should be avoided at factor levels < 1 IU/dL; aspirin may be considered at levels > 5 IU/dL; and individual risk-benefit assessment is required for levels between 1 and 5 IU/dL [27]. High-intensity antithrombotic therapy, such as during PCI for ACS or treatment of acute VTE, should be administered for the shortest effective duration, with factor levels maintained at 80-100 IU/dL throughout [28].

Case resolution: This patient with severe hemophilia A (FVIII baseline 2%, no inhibitors, on rFVIII prophylaxis, not receiving emicizumab) presents with high cardiovascular risk requiring concurrent antiplatelet and antithrombotic management. For ACS, FVIII should be raised to 80-100 IU/dL for at least 48 hours before invasive coronary stratification [27]. DAPT (factor ≥ 30 IU/dL) should be maintained for four weeks post-PCI, followed by indefinite aspirin monotherapy (factor ≥ 5 IU/dL). For AF management, the patient's CHA₂DS₂-VASc score is 3; however, his baseline FVIII activity of 2 IU/dL represents a very high bleeding risk, rendering long-term anticoagulation contraindicated [31]. LAA closure is the most appropriate strategy in this context, in line with current guideline recommendations [28,29]. Post-procedure factor management follows the same principles as perioperative ACS care, with indefinite aspirin therapy maintained at factor levels ≥ 5 IU/dL [29].

Key points

**Figure 1:**
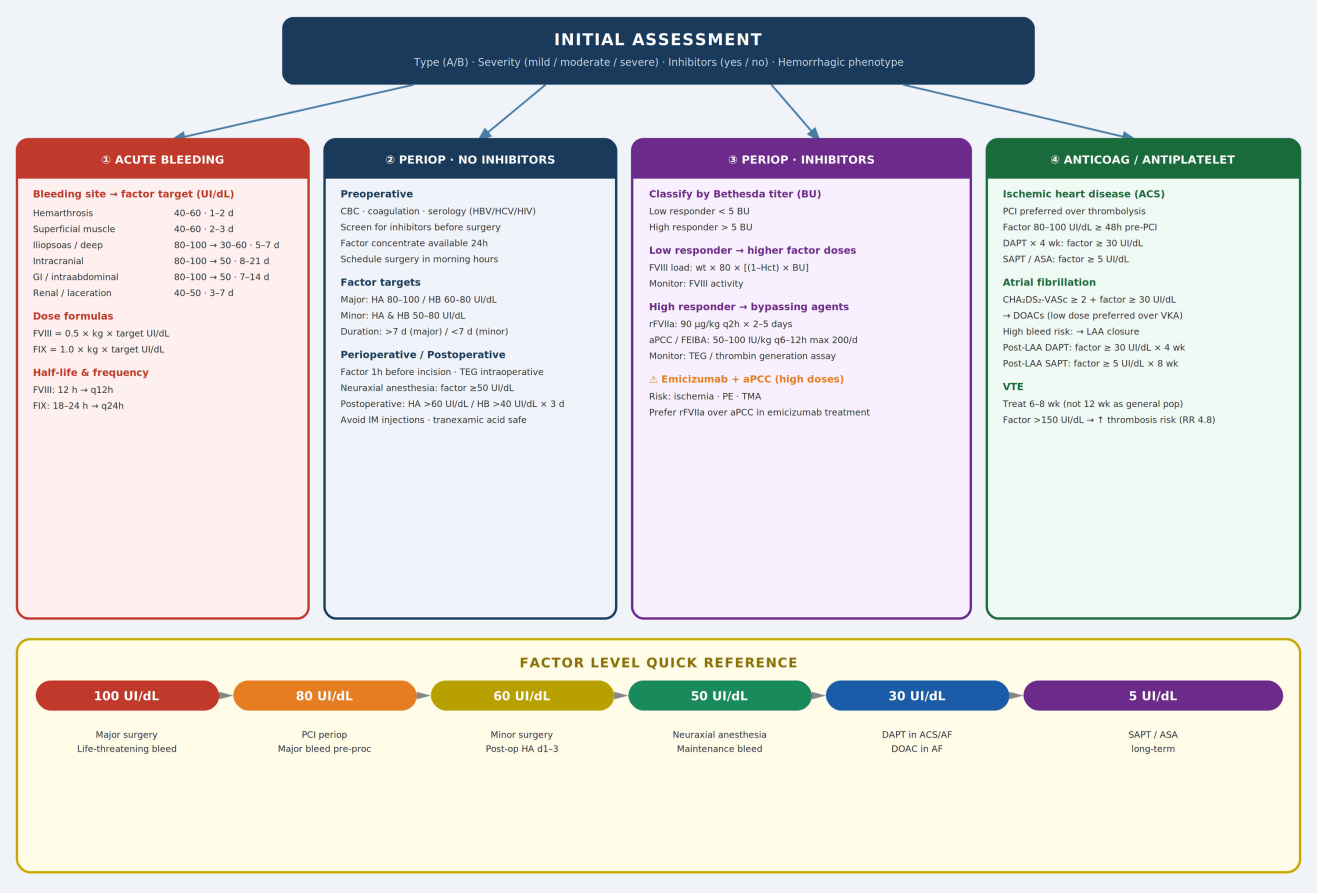
Clinical management algorithm for the hospitalized patient with hemophilia aPCC/FEIBA: Activated prothrombin complex concentrate/Factor eight inhibitor bypassing activity; ACS: Acute coronary syndrome; AF: Atrial fibrillation; ASA: Acetylsalicylic acid (aspirin); BU: Bethesda units; DAPT: Dual antiplatelet therapy (ASA + P2Y12 inhibitor); DOAC: Direct oral anticoagulant; FVIII/FIX: Coagulation factor VIII/Factor IX; HA: Hemophilia A; HB: Hemophilia B; HBV/HCV/HIV: Hepatitis B virus/Hepatitis C virus/Human immunodeficiency virus; LAA: Left atrial appendage; LMWH: Low molecular weight heparin; NSTEMI: Non-ST elevation myocardial infarction; PCI: Percutaneous coronary intervention; PE: Pulmonary embolism; rFVIIa: Recombinant activated factor VII; rFVIII: Recombinant factor VIII; SAPT: Single antiplatelet therapy; TEG/ROTEM: Thromboelastography/Rotational thromboelastometry; TMA: Thrombotic microangiopathy; VKA: Vitamin K antagonist; VTE: Venous thromboembolism. Credit: This image was elaborated by the authors themselves and derived from the clinical scenarios discussed in the present review.

- Key clinical messages derived from the four clinical scenarios discussed in this review are summarized in Figure 1. This figure summarizes the key clinical decisions across the four principal scenarios addressed in this review.

- Hemophilia A and B are secondary hemostasis disorders defined by a deficiency of coagulation factor VIII or IX, respectively. Baseline factor activity determines disease severity - mild (6-40 IU/dL), moderate (1-5 IU/dL), or severe (<1 IU/dL) - and guides bleeding risk stratification [21].

- Factor replacement therapy remains the cornerstone of both prophylactic and on-demand management across all clinical scenarios. In acute bleeding, the anatomical site determines the target factor level, from which the replacement dose is calculated [21].

- All hospitalized patients with hemophilia should be screened for inhibitors and classified as low or high responders, as this distinction is critical for selecting between factor replacement and bypassing agent strategies (recombinant activated factor VII or activated prothrombin complex concentrate) [19].

- Perioperative management, whether in inhibitor-positive or inhibitor-negative patients, requires a multidisciplinary approach, 24-hour laboratory and blood bank availability, and adequate supply of factor concentrates or bypassing agents [15].

- Hemophilia does not confer protection against thromboembolic events. AF and ischemic heart disease occur at rates comparable to the general population in aging patients with hemophilia [29].

- Antithrombotic decisions must be individualized according to baseline factor levels, bleeding phenotype, and inhibitor status. The CHA₂DS₂-VASc score guides anticoagulation in AF; reperfusion therapy for ischemic heart disease requires periprocedural factor levels of 80-100 IU/dL, with targets of ≥30 IU/dL for dual antiplatelet therapy and ≥5 IU/dL for single antiplatelet therapy [29].

## Conclusions

Management of hospitalized patients with hemophilia remains an ongoing challenge that requires adequate resource availability, trained healthcare personnel, and a solid understanding of the major complications and therapeutic approaches associated with this condition. This narrative review, structured around four illustrative clinical scenarios, offers a practical framework for clinicians facing the most common inpatient management decisions: acute bleeding, perioperative care with and without inhibitors, and antithrombotic therapy in the context of cardiovascular comorbidity. As life expectancy improves, clinicians must be equally adept at managing acute bleeding episodes and complex age-related comorbidities. A multidisciplinary, individualized approach - grounded in current evidence and guided by factor levels, inhibitor status, and clinical context - is essential to optimizing outcomes in this population.

The authors acknowledge that, as a narrative review illustrated with fictitious clinical cases, this manuscript does not constitute a systematic appraisal of the evidence. The conclusions are therefore intended to serve as a practical reference for hospital-based clinicians rather than as formal clinical practice recommendations. Future prospective studies and updated clinical guidelines are needed to address the evolving complexity of care in aging patients with hemophilia.
